# Correlates of ever had sex among perinatally HIV-infected adolescents in Uganda

**DOI:** 10.1186/s12978-015-0082-z

**Published:** 2015-10-16

**Authors:** Scovia Nalugo Mbalinda, Noah Kiwanuka, Lars E. Eriksson, Rhoda K. Wanyenze, Dan Kabonge Kaye

**Affiliations:** Department of Nursing, Makerere University, Kampala, Uganda; School of Public Health, Makerere University, Kampala, Uganda; Department of Neurobiology, Care Sciences and Society, Karolinska Institutet, Stockholm, Sweden; Department of Infectious Diseases, Karolinska University Hospital, Stockholm, Sweden; School of Health Sciences, City University London, London, UK; Department of Obstetrics and Gynaecology, Makerere University, Kampala, Uganda

**Keywords:** HIV, Perinatally infected, Adolescents, Risky sexual behaviour, Adolescents, Uganda

## Abstract

**Background:**

The objective of this study was to explore the correlates of ever had sex among perinatally HIV-infected (PHIV) adolescents.

**Methods:**

A cross-sectional survey of sexual behaviour was conducted with 624 PHIV adolescents living three regions (12 districts) of Uganda. Data was collected on socio demographic characteristics (age, sex, occupation, religion and education status), sexual practices and behaviours (Intimate relationships, sexual intercourse, age of sexual debut, condom use, multiple and concurrent sexual partners), consequences of sexual behaviours (pregnancy and STI’s) and life style factors (use of alcohol, psychoactive substances and peer influence). Multivariable logistic-regression was used to ascertain the determinants of sexual activity.

**Results:**

The majority of PHIV were female (59.3 %) and the mean age of the sample was 16.2 (±2.1) years. The mean age of sexual debut was 15.8 years; 16.2 % (101/624) reported symptoms for sexually transmitted infections (STI) and more than a third (213/624) reported ever had sex.Of these 76.5 % (165/213) used condoms inconsistently; and 49.3 % (105/213) had been pregnant or made someone pregnant. Of those in relationships, 56.3 % (223/396) did not disclose and were not aware of their partners’ HIV status. Adolescents aged 15–19 years were more likely to have ever been sexually active (Adjusted odds ratio (AOR) 6.28, 95 % Confidence interval (CI): 2.63-14.99) compared to those aged 10–14 years. Adolescents who were living alone were more likely to have ever been sexually active compared to those living with one or both parents (AOR 4.33, 95 % CI: 1.13-16.62). The odds of being sexually active were lower among adolescents in school compared to those out of school (AOR 0.2, 95 % CI: 0.13-0.30), who had never been treated for STI (compared to those who had never been treated for STI) (AOR 0.19, 95 % 0.11-0.32) and adolescents who never drank alcohol (AOR 0.49, 95 % CI 0.28-0.87).

**Conclusion:**

PHIV adolescents have risky sexual behaviours characterized by being sexually active, inconsistent condom use, and having partners of unknown status. Risk reduction interventions are required to minimize unplanned pregnancies, STI, and HIV transmission by PHIV adolescents.

## Background

The global paediatric HIV epidemic has shifted into a new phase as children on antiretroviral therapy (ART) grow into adolescence and adulthood, where they face new challenges of living with HIV [1]. Like other adolescents, perinatally HIV-infected adolescents (PHIV) experience challenges related to their stage of development as they enter puberty [2]. The PHIV mature physiologically and psychologically and become sexually active. Initiation of sexual activity during adolescence among PHIV predisposes them to risk of pregnancy and STI’sincluding acquiring other strains of HIV and infecting other adolescents which compounds the HIV infection rates [2, 3].

Globally, it is estimated that about 2.1 million adolescents (10–19 years) are HIV positive [4]. Most of these adolescents acquired HIV perinatally from their HIV infected mothers during pregnancy, birth or when they were breastfed. Nearly 2 million of these adolescents live in sub-Saharan Africa: 70 % of whom live in South Africa, Nigeria, Kenya, Tanzania, Uganda, Zimbabwe, Mozambique, Zambia, Ethiopia and Malawi [5]. For instance, in the year 2012, about 260,000 new HIV infections occurred in children worldwide [6]. In Uganda, HIV prevalence has been rising since its lowest rate of 6.4 percent in 2006 [7]. Currently, 7.3 percent of Uganda’s population is living with HIV [8]. New infections are diagnosed in 150,000 people each year, of whom 20,600 are children [7]. In addition, in 2012, 110,000 adolescents were living with HIV in Uganda [9]. The HIV prevalence in adolescents aged 15–17 years is 1.6 % in females and 1.8 in males, while among adolescents aged 18–19 years, the prevalence is 5.1 % in females and 1.5 % in males [10]. In Uganda, data on whether these adolescents acquired HIV perinatally or behaviorally is not known. Understanding the sexual behaviors of PHIV can help us can help us explain some of the factors related the HIV infection rates in this population.

Current literature based on studies conducted in developed countries and focusing on sexual behaviours of adolescents and young people who acquired HIV behaviourally or perinatally highlights differences in risky sexual behaviours [11–14]. This data suggests that irrespective of how HIV was acquired, adolescents have high-risk behaviour characterised by having unprotected sex and multiple sexual partners [15]. There are various factors which have been found to be responsible for increasing the risk of being sexually active among adolescents. Contextual factors such as the use of alcohol or marijuana, living environment, caregivers’ mental health increase the chances of engaging in penetrative sex among the youth [16]. In addition, living with only one or with neither parent or having low parental supervision increased the frequency of protected and unprotected sex [17]. Furthermore, not receiving instructions at school about pregnancy prevention increased the frequency of protected and unprotected sex [17].

Many PHIV adolescents are actively engaged in sexual activity and report prior or current sexual experience [14, 18]. A study conducted in Uganda showed that 11.4 % of females and 12.9 % of males had their sexual debut between the ages of 15–19 years [19]. To reduce the risk of HIV transmission or acquisition of other HIV strains (super infection), the main focus of existing HIV programs is to encourage young clients with HIV infection to refrain from or postpone sexual activity [18, 20]. Yet from sexual and reproductive health (SRH) and rights, adolescents need to be given adequate information so as to make appropriate decisions concerning their sexual and reproductive health.

The data available on sexual behaviour of HIV positive adolescents in Uganda has several shortfalls. It is mainly from qualitative studies and with few quantitative studies. Secondly, most of the data was collected from the central region in Uganda. This necessitates studies in other regions of the country to compare findings and as well as studies that use both qualitative and quantitative methods. In addition, the available data is not stratified among younger (10–14 years old) and older (15–19 years old). Moreover, the data does not segregate information from adolescents perinatally infected with HIV and those who acquired HIV through other means. Lastly, correlates and contextual factors of being sexually active in HIV perinatally-infected adolescents are not well documented.

In Uganda, the proportion and correlates of being sexually active among PHIV adolescent is not known. This curtails effective planning of potential health services for this population. The purpose of the current study was to describe the correlates of being sexual active among perinatally HIV-infected adolescents. This was a part of larger study that looked at sexual experiences, quality of care of SRH services and health related quality of life among PHIV adolescents.

## Methods

### Study design and setting

This was a cross sectional study of PHIV adolescents aged 10–19 years receiving care and treatment in12 antiretroviral therapy (ART) clinics in three regions of Uganda (Eastern, Western and Northern). Data was collected from September 1, 2013 to March 30, 2014.

### Participant’s selection

Three regions (Northern, Eastern and Western) were purposefully-selected. The central region was excluded because previous studies on PHIV adolescents were conducted in this region. In each region, four health facilities were selected. The regional referral hospitals were purposefully-selected from each region because they receive referrals from the lower level facilities. To select the remaining facilities, a sampling frame comprising of all health facilities in each region with 50 or more clients aged <15 years were considered [21]. Three health facilities were then randomly selected from each of the regions.

The inclusion criteria for participants were being adolescents aged 10–19 years and knew their HIV sero-status. At each facility the participants who met the inclusion criteria and consented to participate in the study were selected through a consecutive sampling procedure. At each site, a research assistant recruited all the participants who were available in the ART clinic, and enrolled those who fulfilled the inclusion criteria and gave informed consent for participation, until the sample size was obtained. To verify and confirm PHIV status of the participants, medical records were checked to ascertain the HIV positive result, a positive test from parents, and absence of information that HIV was acquired sexually or through injections. The age at which the adolescents started attending the ART clinic was also reviewed and recorded to corroborate the PHIV status and possible source of infection. The research assistants were trained on interview skills and on how to ensure privacy and confidentiality. Trained research assistants conducted the interviewed the the participants in absence of their parents or guardians.

### Sample size determination

The sample size was powered to determine the prevalence of being sexually active. The sample size was calculated using a prevalence of 33 % of ever having sex, 95 % confidence interval and an error margin of 5 %. The prevalence of 33 % was used based on a previous study by Birungi et al. [22]. A design effect of 2 was used and we recruited 624.

### Variable measurements

Data were collected through face to face interviewer- administered questionnaires in a private room. The data collected from participants included socio-demographic characteristics (age at last birthday, gender, education level, religion marital status, living situation and occupation), sexual behaviour and practices (“having ever had sex”, boyfriend/girlfriend, and intimate relationships), condom use (use of condom in the last 6 months and correct and consistent condom use every time the adolescent had sex), number of concurrent sexual partners, STI’s, disclosure of HIV sero-status to the partner; pregnancy and procreation intentions; and peer influence. The primary outcome measured was having ever had sex, which was defined as heterosexual penetrative intercourse. Data on sexual experiences among adolescents was obtained using a series of validated questionnaires adopted from a number of tools [19, 23]. The tools were piloted to remove ambiguity by reframing and rephrasing some questions.

### Data management and analysis

Participant’s age was grouped into 10–14 years and 15–19 years. Education status was grouped into in school and out of school, and education level was grouped into three categories: no education, primary and secondary. Occupation was grouped into three categories: students, unemployed and employed.

The proportion of those who had ever had sex was computed. To assess correlates of having ever had sex as the dependent variable and other variables, Pearson’s chi square and Student t test were used to measure the association between these variables and for categorical and continuous explanatory variables respectively.

Stepwise logistic regression models were built to identify independent predictors of ever had sex. During model development, all predictor variables with a p-value of ≤0.2 [24] at bivariate analysis were considered for inclusion in the multivariate logistic regression model. Collinearity was assessed using a correlation matrix and cross-checked by the use of variance inflation factor which was set at 10 [25]. In case two variables were associated (*P* < 0.05), the variable explaining the largest variability (smaller p value at univariate analysis) was retained. Significance was set at 0.05 and all of the analyses were two tailed. Analyses were done using STATA®12.

### Ethical review and approval

Ethical reviews and approval were obtained from the Higher Degrees and Research Ethics Committee of the College of Health Sciences at Makerere University and the Uganda National Council for Science and Technology. Administrative clearance and permissions were also obtained from the management of each of the health facilities. Written informed consent was obtained from adolescents above 18 years. For adolescents below 18 years assent from the adolescents and consent from parents or guardians was obtained. All the participants who were in need of services such as family planning or screening for Sexually Transmitted Infections (STI’s) were offered these services by referral. Participation was voluntary and all the interviews were conducted in private settings to ensure participant’s confidentiality.

## Results

A total of 624 perinatally HIV-infected adolescents participated in the study and their characteristics are presented in Table 1. Of these, 370/624 (59 %) were female, 458/624 (73.4 %) were in school at the time of the study and 356/624 had primary level education (57.6 %). The mean age of the participants was 16.2 years (SD 2.1) and mean age of sexual debut was 15.8 years. The mean age at which the PHIV adolescents started attending the ART clinic was 6.2 years (SD 2.0).

**Table 1:** Socio demographic characteristics of 624 perinatally HIV -infected adolescent in Uganda

Characteristics		Frequency (n)	Percent (%)
Gender	Females	370	59.3
	Males	254	40.7
Age groups	10 – 14 years	114	18.3
	15 – 19 years	510	81.4
Religion^a^	Catholic	260	41.8
	Protestant	252	40.4
	Muslim	44	7.1
	Born again	58	9.3
	Other	7	1.1
Education status	Out of school	166	26.6
	In school	458	73.4
Level of education^a^	No formal education	30	4.8
	Primary level	356	57.1
	Secondary level	231	37.1
Highest level of education hoped to complete	Primary	12	2.6
	Secondary	40	8.7
	Tertiary	378	82.4
	Did not know	28	6.1
Occupation^a^	Students	462	74
	Employed	49	7.9
	employed	86	13.7
Region	Western	231	37
	Eastern	173	27.7
	Northern	220	35.3
Distance to clinic	Within 5 km	197	31.5
	More than 5 km	427	68.6
Parenthood status (living status)^a^	Both parents	105	16.8
	One parent (one dead)	196	31.4
	One parent (other alive)	51	8.2
	Guardian/siblings (parents dead)	245	39.3
	Staying alone	19	3.0

^a^Some responses were missing/unknown

Table 2 shows the sexual behaviours and other behaviours of the participants.. Of the 624, 519/624 (83 %) were currently on ARV’s, 396/624 (64 %) had ever been in an intimate relationship, 206/624 (33 %) were currently in an intimate relationship and. 213 (34.%) had ever had sexual intercourse. Of the latter, 144/213 (57 %) were female, 136/213 (64 %) had sex in the last six months, 93/213 (44 %) did not use condoms during the last sexual encounter, 165/213 (76 %) did not use condoms consistently, and 105/213 (49 %) had been pregnant or made someone pregnant. In regard to STI’s 101/624 (16 %) reported having related symptoms. Of these, 65/101 (64 %) had advised their partners to seek care.

**Table 2:** Sexual and other behaviours of perinatally HIV- infected adolescents with HIV in Uganda

Variable	Total (N)		n(%)
Currently on ARVs	624	Yes	519 (83.2)
Ever had Intimate relationship	624	Yes	396 (63.5)
Currently in intimate relationship	624	Yes	206 (33.0)
Ever had sex	624	Yes	213 (34.1)
Had sex in the last 6 months?	213	Yes	136 (63.9)
^b^How often has sex:	136	≥3 times a week	17 (12.5)
		Once week	41 (30.2)
		Once a month	78 (57.4)
Number of sexual partners(last six months)	213	1	179 (84.0)
		More than 1 partner	34 (16.0)
^a^Used a condom at last sex	213	No	93 (43.7)
^a^Consistent condom use	213	No	165 (77.5)
^c^Ever been pregnant	144	Yes	82 (56.9)
^c^Ever made someone pregnant	69	Yes	23 (33.3)
Aware of HIV status of partner and disclosed	396	No	223 (56.3)
Partner HIV +	173	Yes	86 (49.7)
Disclosed HIV status	396	No	196 (49.5 %)
^a^Ever lived with boy/girlfriend?	213	Yes	95 (44.6)
^a^Ever been married?	213	Yes	46 (21.6)
Sexually transmitted infections			
Been treated for an STD/STI?	624	Yes	101 (16.2)
Ever suffered from the following symptoms	101	Genital sores	15 (14.8)
		Genital itching	28 (27.7)
		Genital discharge	11 (10.9)
		Lower abdominal pain	17 (16.8)
Advised partner to seek treatment	101	Yes	65 (61.9)
Peer influence	N		n (%)
Ever smoked cigarette?	624	Yes	18 (2.9)
Ever drunk alcohol?	624	Yes	81 (12.0)
Has any friend taking alcohol?	624	Yes	92 (14.7)
Ever been Influenced to smoke/take alcohol	624	Yes	110 (17.6)
Sexuality	N		Mean (SD)
Age when had first boy/girlfriend (years)	396		15.67 (1.5)
^a^Age when had first sex (years)	213		15.83 (1.7)
Age when got married (years)	46		17.30 (1.1)

An intimate relationship is an interpersonal relationship that involves physical or emotional intimacy. Physical intimacy is characterized by romantic or passionate attachment or sexual activity

Boyfriend and girlfriend means a regular female/ male companion with whom a person has a romantic as well as sexual relationship

^a^These refer to those 213 had ever had sex

^b^These refer to those 136 who had sex in the last six months

^c^These refer to the females or males who had ever been sexually active

Of the 624 adolescents, 396/624 (63.5 %) adolescents had ever been in relationships, of whom 196/396 (50 %) had not disclosed their sero status to their partner, and 173/396 (44 %) were aware of the HIV status of their partner. For the latter, 86/173 (50 %) had HIV positive partners. About lifestyle, 15.9 % (99/624) had ever drunk alcohol and/ smoked.

Table 3 shows stratified analysis comparing young and old adolescent’s sexual behaviour. PHIV adolescents aged 10–14 years had ever had sex (2.8 % vs 97.2 %; p < 0.001) compared to those aged 15–19 years. About 3 % (6/213) of adolescents aged 10–14 years and 22/213 (10 %) of adolescent aged 15–19 years had their first sex before the age of 15. In regard to condom use at last sex, adolescents aged 10–14, 3/120 (2.5 %) had used condoms compared to 117/120 (97.5 %) adolescents aged 15–19 years. None of the adolescents aged 10–14 had more than one sexual partner. However, 34/213 (16 %) of the adolescents who had ever had sex had more than one concurrent sexual partner.

**Table 3:** Stratified analysis comparing young adolescents and older adolescent’s sexual behaviour among perinatally HIV-infected adolescents

Variables	10-14 years N (%)	15-19 years N (%)	Total
Ever had sex			
Yes	6 (2.8)	207 (97.2)	213 (100)
No	108 (27.3)	303 (73.7)	411 (100)
Age at first sex			
10-14	6 (21.4)	22 (78.6)	28 (100)
15-19	-	185 (100)	185 (100)
Condom use at last sex			
Yes	3 (2.5)	117 (97.5)	120 (100)
No	3 (3.3)	90 (96.7)	93 (100)
Number of partners			
One	6 (3.4)	173 (96.6)	179 (100)
>one partner	-	34 (100)	34 (100)

### Factors associated with ever having had sex

The factors associated with ever having had sex among PHIV are presented in Tables 4 and 5. The results show that female gender (OR = 1.71, 95 % CI =1.21-2.42), age group of 15–19 years, (OR =12.38, 95 % C I = 5.34-28.7), and living alone (OR = 5.37, 95 % CI 1.79-16.09), were significantly associated with ever having sexual intercourse. On the other hand being in school (OR = 0.14, 95 % CI = 0.10 – 0.21), receipt of STI treatment (OR = 0.14, 95 % CI 0.09 – 0.23), not drinking alcohol (OR = 0.38, 95 % CI (0.24 – 0.61), not smoking (OR = 0.10, 95 % CI =0.03 – 0.34), and not having a friend who drinks or smokes decreased the risk of ever having had sex OR = 0.30, 95 % CI (0.19-0.47). And as shown in Table 6, the independent predictors of being in this sample were; age group of 15–19 years (AOR = 6.28, 95 % CI =95 % 2.63 – 14.99), being in school, (AOR = 0.20, 95 % CI = 0.13-0.30), living alone, (AOR = 4.3, 95 % CI = 1.13-16.62), not ever been treated for STI (AOR 0.19 95 % CI 0.11-0.32), and not ever drunk alcohol (AOR = 0.49, 95 % CI 0.28-0.87).

**Table 4:** Likelihood of ever having had sex by socio demographic factor for perinatally HIV-infected adolescents in Uganda (2013/2014)

Variables		Sexually active		Unadjusted	*P*-value*
		Yes N (%)	No N (%)	OR (95 % CI)	
Sex	Male	69 (32.39)	185 (45.01)	1	0.001
	Female	144 (67.61)	226 (54.99)	1.71 (1.21 – 2.42)	
Age groups	10 – 14 years	6 (2.82)	108 (26.41)	1	0.001
	15 – 19 years	207 (97.18)	301 (73.59)	12.38 (5.34-28.70)	
Religion	Catholic	88 (41.90)	172 (41.85)	1	0.83
	Protestant	83 (39.52)	169 (41.12)	0.96 (0.66 – 1.39)	0.23
	Muslim	19 (9.05)	25 (6.08)	1.49 (0.78 – 2.84)	0.51
	Born again	17 (8.10)	41 (9.98)	0.81 (0.44 – 1.51)	
Education status	Out of school	110 (51.64)	52(12.65)	1	0.001
	In school	103 (47.89)	356 (86.62)	0.14 (0.10 – 0.21)	
Level of education	None	13 (6.10)	17 (4.21)	1	0.15
	Primary level	109 (51.17)	247 (61.14)	0.58 (0.27 – 1.23)	0.68
	Secondary level	91 (42.72)	140 (34.65)	0.85 (0.39 – 1.83)	
Education level hoped to complete	Primary	30 (17.96)	35 (9.07)	1	0.13
	Secondary	38 (22.75)	72 (18.65)	0.62 (0.33 – 1.15)	0.001
	Tertiary	99 (59.28)	279 (72.28)	0.41 (0.24 – 0.71)	
Occupation	Students	102 (52.04)	360 (89.78)	1	0.001
	Volunteers(employed)	33 (16.84)	16 (3.99)	7.28 (3.85-13.75)	0.001
	Stay home	61 (31.12)	25 (6.23)	8.61 (5.15- 14.41)	
Region	Western	65 (30.52)	166 (40.39)	1	0.001
	Eastern	72 (33.80)	101 (24.57)	1.82 (1.20 – 2.76)	0.14
	Northern	76 (35.68)	144 (35.04)	1.35 (0.90 – 2.01)	
Living situation	Both parents	36 (17.39)	69 (16.87)	1	0.52
	One parent (one dead)	60 (28.99)	136 (33.25)	0.85 (0.51 – 1.40)	0.72
	One parent (other alive)	19 (9.18)	32 (7.82)	1.14 (0.57 – 2.28)	0.65
		78 (37.68)	167 (40.83)	0.90 (0.55 – 1.45)	

*Fisher’s exact test was used where we had a cell count less than 5

**Table 5:** Likelihood of medical and life style factors associated with ever having had sex among perinatally HIV -infected adolescents

Variables		Sexually active		Unadjusted	*P*-value*
		Yes N (%)	No N (%)	OR (95 % CI)	
Ever been treated for an STD/STI	Yes	73 (34.43)	28 (6.86)	1	0.001
	No	139 (65.57)	380 (93.14)	7.1 (4.42 – 11.48)	
Ever smoked cigarette	Yes	15 (7.08)	3 (0.78)	1	0.001
	No	197 (92.92)	408 (99.27)	0.10 (0.03 – 0.34)	
Ever drunk alcohol	Yes	44 (20.66)	37 (9.00)	1	0.001
	No	169 (79.34)	374 (91.00)	0.38 (0.24 – 0.61)	
Has a friend who is smoking cigarette	Yes	54 (25.47)	38 (9.29)	1	0.001
	No	158 (74.53)	371 (90.71)	0.30 (0.19 – 0.47)	
Has a friend who is drinking alcohol	Yes	100 (47.17)	85 (20.68)	1	0.001
	No	112 (52.83)	326 (79.32)	0.29 (0.20 – 0.42)	
Ever been influenced to smoke/drink	Yes	67 (31.90)	43 (10.49)	1	0.001
	No	143 (68.10)	367 (89.51)	0.25 (0.16 – 0.38)	

*Fisher’s exact test was used where we had a cell count less than 5

**Table 6:** Adjusted effects for predictors of ever having had sex from a multiple logistic regression among perinatally HIV -infected adolescents

	Sexually active			
Variables	Yes num. (%)	No num. (%)	Adjusted OR (95 % CI)	P-Value
Age groups				
10 – 14 years	6 (2.82)	108 (26.41)	1	0.001
15 – 19 years	207 (97.18)	301 (73.59)	6.28 (2.63 – 14.99)	
Education status				0.001
Out of school	111 (52.11)	55 (13.38)	1	
In school	102 (47.89)	356 (86.62)	0.20 (0.13 – 0.30)	
Parenthood status - Living with				
Both parents	36 (17.39)	69 (16.87)	1	0.68
One parent (one dead)	60 (28.99)	136 (33.25)	0.88 (0.48 – 1.61)	0.78
One parent (other alive)	19 (9.18)	32 (7.82)	1.13 (0.47 – 2.69)	0.61
Guardian/siblings	78 (37.68)	167 (40.83)	0.86 (0.48 – 1.54)	0.03
Living alone	14 (6.76)	5 (1.22)	4.33 (1.13 – 16.62)	
Ever been treated for an STD/STI				
Yes	73 (34.43)	28 (6.86)	1	0.001
No	139 (65.57)	380 (93.14)	0.19 (0.11 – 0.32)	
Ever drunk alcohol				
Yes	44 (20.66)	37 (9.00)	1	0.02
No	169 (79.34)	374 (91.00)	0.49 (0.28 –0.87)	

*AOR* adjusted odds ratio, adjusted for variables included in the model

## Discussion

The aim of this of this paper was to investigate the the correlates of ever having had sex among PHIV adolescents. The findings of the study show that one in three PHIV adolescents had ever had sex and the correlates of ever having had sex were age, school status, living alone without parents and alcohol use. . About 16 per cent engaged in risky sexual behaviours characterised by multiple, concurrent partners and are at risk of HIV re-infection, pregnancy and STIs.

Sexual experiences are a sensitive matter. Collecting data on sexual experiences is could lead socially desirable responses. We used several measures to address this: We assured adolescents of confidentiality by conducting the interview in a private room; permission was sought from the adolescents, they were informed about the importance of the study and were assured that everything discussed in the session would be kept confidential and would not be shared with the health providers or their parent. We asked sensitive questions in many different ways. All the questions were answered with no skips; data was collected by research assistants who are not part of the clinic staff.

Adolescents’ risky sexual relationships put them at risk of HIV re-infection, pregnancy and STIs. About a third of PHIV adolescents who participated in the study reported that they had ever had sex, with some reporting having unprotected sex .If the adolescents don’t know the sero status of their partners they can infect them with HIV and also be re-infected. If they are not adhering to ART, the females can get pregnant and pass on HIV infection to their new-borns. It is apparent that the PHIV adolescents interviewed either do not have knowledge about the dangers of having unprotected sex or if they do, have not appreciated the dangers involved and are not adhering to the safer sexual practices they have been taught. According to the SRH and rights framework, adolescents need to be given adequate information so as to make appropriate decisions concerning their sexual and reproductive health. Regardless of their HIV status, they have the freedom to choose partners and even have sexual relationships, but in compliance with the laws of the country. They also have the responsibility to avoid, prevent or mitigate adverse effects of their sexual behaviours including re-infection and resultant HIV drugs resistance. They thus need to have adequate information and support to make informed decisions about their sexuality and sexual and reproductive health. This includes information on safe sexual practices and behaviours and contraception. Generally these findings are consistent with those of other studies done in Uganda, and in developed and less developed countries which have indicated that PHIV adolescents begin to explore their sexuality by dating and getting involved in relationships by 10–14 years [26–30]. A higher proportion of PHIV adolescents in this study were sexually active (34 % versus 12 % of the adolescents who had sex intercourse before age 15 in the general population [10].

The pregnancy intentions of HIV-positive clients are driven not only by an HIV diagnosis but by individual concerns as well as larger societal and cultural expectations [31]. In the current study PHIV adolescents who reported having ever had sex, 49 % had ever been pregnant or impregnated. A number of the PHIV adolescents reporting pregnancy had been in care for a long time as shown by the mean age at which they joined care. Whereas it is assumed that since these adolescents have been in care for a long time they are exposed to many sexual and reproductive messages this may not be the case. Access to SRH messages depends on the quality of SRH services in ART clinics or may depend on whether the adolescents are from urban or rural area [32]. Therefore by being sexually active, PHIV adolescents are at risk of early pregnancy, contracting STIs and re-infection; and others at risk of contracting HIV/STIs. The findings implies that the current strategies and health programs used for health education and to stop transmission of HIV specifically those targeting PHIV adolescents need to be re-visited because a large number are sexually active or are not having protected sex.

The findings of the current study also show that correct and consistent condom use was low (22 %) compared to the general population which stands at 25.5 % and 31.7 % for females and males respectively [8]. Findings of prior studies revealed that for HIV positive adolescents who were sexually active, many were not consistently using condoms [15, 33]. Another study observed that PHIV adolescents are less likely to consistently use condoms compared to those who are HIV negative [34]. The fact that most adolescents who were in relationships did not know their partners’ sero status or disclose their sero status to their partners implies increased risk of HIV transmission or re-infection in/by sexually active PHIV adolescents. Studies have also found that PHIV adolescents engage in risky sexual behaviours even when they know their status [27, 35–37].

Our study suggests that one of the determinants of sexual activity among PHIV adolescents is peer influence. Peer influence informs the normative or acceptable behaviour for certain ages of adolescents [2, 38]. Peer influence leads to adoption a particular type of behaviour, dress, or attitude in order to be accepted as part of a group of their equals ("peers"). Adolescents' sexual behaviours are influenced by the sexual attitudes and behaviours of their friends [39, 40]. This is consistent with other studies that have shown that youths who had friends who were sexually experienced have greater odds of early sexual debut [2, 39, 41]. In our study PHIV adolescents who consumed alcohol and smoked cigarettes were more likely to have ever had sex. Other studies have also reported similar findings and these show that adolescents' use of alcohol and other drugs has been associated with sexual risk [42–44]. Alcohol compromises the decision-making or leads to disinhibition when adolescents are drunk, and this adds another dimension to the increased behavioral risks of PHIV adolescents [45].

Schools can be a primary source of information about prevention methods in the fight against HIV [46]. This study has shown that being in school was associated with lower odds ofever having had sex. Education also accelerates behaviour change among young men and women by making them more receptive to prevention messages [47]. Schools also provide adults and young people with the life skills they need to make informed choices and to develop both economic and intellectual independence [47]. Studies have shown that increasing number of years are associated with a less likelihood of having a casual sexual partner and a high likelihood of condom use [48]. It has also been already reported in other countries that better-education for girls tended to delay having sex and increased instance on condoms use in their partners [22, 49].

Living arrangements of PHIV adolescent and availability of parental guidance is another key factor that determines the adolescent is sexually active or not among PHIV adolescents. In our study living alone was independently associated with ever having had sex. This was not surprising because adolescents living alone have no guidance from elders, parents or siblings [50]. Therefore they luck in social support and social skills and it is very easy for them to become more vulnerable to peer influence and subsequent risky sexual behaviours. Studies have shown that living alone or living with siblings is associated with risky sexual behaviours compared to living with older people such as grandparents, parents or guardians [15, 16, 51, 52].

### Limitations of the study

The study had several limitations emerging from the self-report method of data collection such as the recall bias (recall of sexual activity) and the potential for under-reporting of sexual behaviour due to the sensitive nature of the subject. Participants may have forgotten or may have been embarrassed to reveal information their sexual activity especially for the females.The males may have exaggerated their sexuality activities (20). In addition, it is possible that the participants may have forgotten important information on issues like condom use and number of partners. On participant selection, information was not available on the mother’s HIV status; this could have introduced some misclassification bias. We however collected information on age at which adolescents started visiting ART clinic and we used this as a proxy of perinatal infection. Furthermore, data on HAART was not collected and this might have added valuable relevancy since treatment improves health and quality of life, and likelihood of forming relationships and being sexually active. Testing for STIs and monitoring of HIV parameters such as CD4 count and viral load were not part of the study. These measurements are critically linked to sexual behaviour in people who are on ART [53, 54].

## Conclusion and implication

Perinatally HIV-infected (PHIV) adolescents in Uganda are sexually active and engage in risky sexual behaviours such as having unprotected sex intercourse. The majority of the PHIV adolescents do not know the sero-status of their partners and do not use condoms consistently. The level of ever having had sex and risky sexual behaviours increases the risk of HIV transmission and HIV re-infections among the sexual partners of PHIV adolescents. The key correlates of ever having had sex among PHIV adolescents are age, school status, and parenthood status and alcohol consumption. There is need for concerted efforts and interventions to ensure that PHIV adolescents stay in school have a stable family support and receive effective sexual and reproductive health educations that reduces risky sexual behaviours and substance abuse.
